# Brassinin Represses Invasive Potential of Lung Carcinoma Cells through Deactivation of PI3K/Akt/mTOR Signaling Cascade

**DOI:** 10.3390/molecules24081584

**Published:** 2019-04-22

**Authors:** Min Hee Yang, Jong Hyun Lee, Jeong-Hyeon Ko, Sang Hoon Jung, Gautam Sethi, Kwang Seok Ahn

**Affiliations:** 1 KHU-KIST Department of Converging Science and Technology, Kyung Hee University, Seoul 02447, Korea; didmini@naver.com (M.H.Y.); shjung507@gmail.com (S.H.J.); 2 Department of Science in Korean Medicine, Kyung Hee University, 24 Kyungheedae-ro, Dongdaemun-gu, Seoul 02447, Korea; mirue88@nate.com (J.H.L.); gokjh1647@gmail.com (J.-H.K.); 3 Department of Pharmacology, Yong Loo Lin School of Medicine, National University of Singapore, Singapore 117600, Singapore

**Keywords:** brassinin, EMT, lung carcinoma, PI3K/Akt/mTOR

## Abstract

The epithelial–mesenchymal transition (EMT) is a phenomenon that facilitates epithelial cells to acquire invasive potential to induce the initiation the metastatic spread of tumor cells. Here, we determined if brassinin (BSN) can affect the EMT process and deciphered its anti-cancer effects. BSN attenuated the levels of EMT linked genes and suppressed transforming growth factor beta (TGF-β)-mediated regulation of diverse mesenchymal markers. Additionally, BSN did increase the expression of various epithelial marker proteins in lung cancer cells. TGF-β-induced morphological changes and induction of invasive ability of tumor cells was also found to be abrogated by BSN treatment. Finally, BSN not only suppressed constitutive, but also inducible phosphoinositide-3-kinase (PI3K)/protein kinase B (Akt)/mammalian target of rapamycin (mTOR) phosphorylation in tumor cells.

## 1. Introduction

Lung carcinoma is a malignant tumor often characterized by abnormal growth and metastasis [1]. The aberrant growth of tumor cells can extend outside the lungs by the process of metastasis and invasion of neighboring tissues or distant organs within the body [2,3,4,5,6]. Primary lung tumors can generally metastasize to the brain, bones, liver, and adrenal glands [7]. It has been found that metastasis rather than primary tumor accounts for the majority of fatalities among cancer patients [8,9,10,11].

The epithelial-to-mesenchymal transition (EMT), although required for various physiological processes, can also contribute pathologically to fibrosis and cancer progression [12,13,14]. Even though EMT is usually tranquilized in adulthood, it can promote transformation of tumor cells to an invasive and metastatic phenotype in lung cancer once stimulated by the growth factor signaling pathway [2]. Generally, during the EMT process, an augmentation of levels of mesenchymal markers, and down modulation of epithelial markers, is frequently encountered [15,16,17].

Various indole phytoalexins, identified from cruciferous (Brassica) vegetables, are important source of anti-neoplastic agents because they can exert several biological activities, such as anti-proliferative and anti-carcinogenic activities [18,19,20]. Among these phytoalexins, brassinin (BSN) was isolated from Chinese cabbage [21] and was found to suppress TNFα-induced vascular inflammation via modulation of reactive oxygen species (ROS) production in endothelial cells [22]. Its derivative, homobrassinin, has also been reported to cause ROS-dependent apoptosis in colorectal cancer cells [23]. Our group has also reported that BSN can inhibit STAT3 signaling cascades through multiple molecular mechanism(s) and also attenuate tumor growth under in vivo settings [24]. Our previous report has also indicated that BSN induced apoptosis by affecting Akt activation in prostate cancer cells [25]. The combination of BSN and capsaicin can exhibit synergistic apoptotic and anti-metastatic activities in prostate cancer cells [26]. However, there are no major studies regarding the actions of BSN on the modulation of EMT biomarkers, therefore this project aimed to decipher the effect of this phytoalexin on constitutive and inducible EMT processes in malignant cells.

## 2. Results

### 2.1. BSN Modulates EMT in Tumor Cells

**Figure 1:**
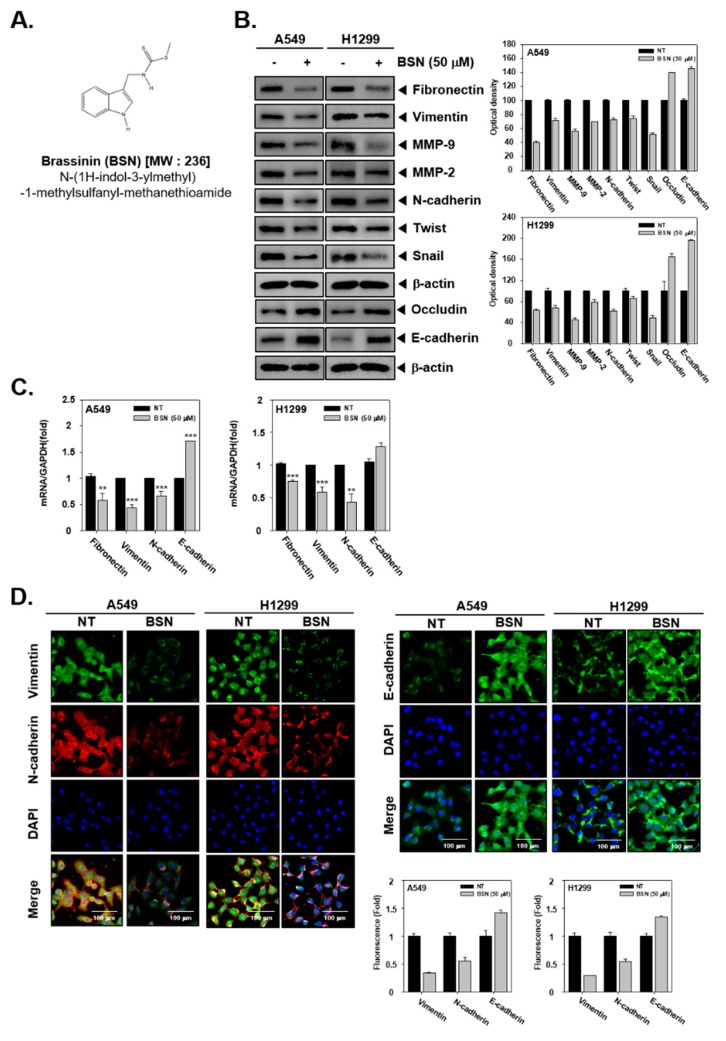
Effects of brassinin (BSN) on epithelial-mesenchymal transition (EMT) in tumor cells. (**A**) Chemical structure of brassinin (BSN). (**B**) Cells (5 × 10^5^/well) were treated with BSN 50 μM (+) for 24 h, and compared with non-treated cells (−). Western blotting for different proteins was thereafter performed. The results shown are representative data of experiments. Graphs represent band intensities of indicated proteins. (**C**) A549 and H1299 cells were treated with BSN (50 μM) for 24 h. The expression of various genes was studied by real-time quantitative polymerase chain reaction (PCR). Data represent means ± SD; *** *p* < 0.001 vs. non-treated (NT) cells, and ** *p* < 0.01 vs. non-treated (NT) cells. (**D**) Cells were treated as indicated before and levels of EMT markers were determined by immunocytochemistry. Quantitative analysis of the fluorescence intensities was performed.

First, we investigated the levels of EMT markers by Western blot analysis. BSN downregulated the levels of fibronectin, vimentin, MMP-9, MMP-2, N-cadherin, Twist, and Snail proteins (Figure 1B). However, it was found that BSN upregulated occludin and E-cadherin expression (Figure 1B). Moreover, we determined the levels of EMT markers by real-time quantitative PCR analysis. BSN reduced the mRNA level of fibronectin and vimentin and enhanced that of E-cadherin (Figure 1C). Furthermore, as observed by immunocytochemistry, drug treatment caused a decrease of vimentin and N-cadherin levels, as well an increase of the E-cadherin level (Figure 1D).

### 2.2. BSN Regulates TGF-β-Induced EMT in Malignant Cells

**Figure 2:**
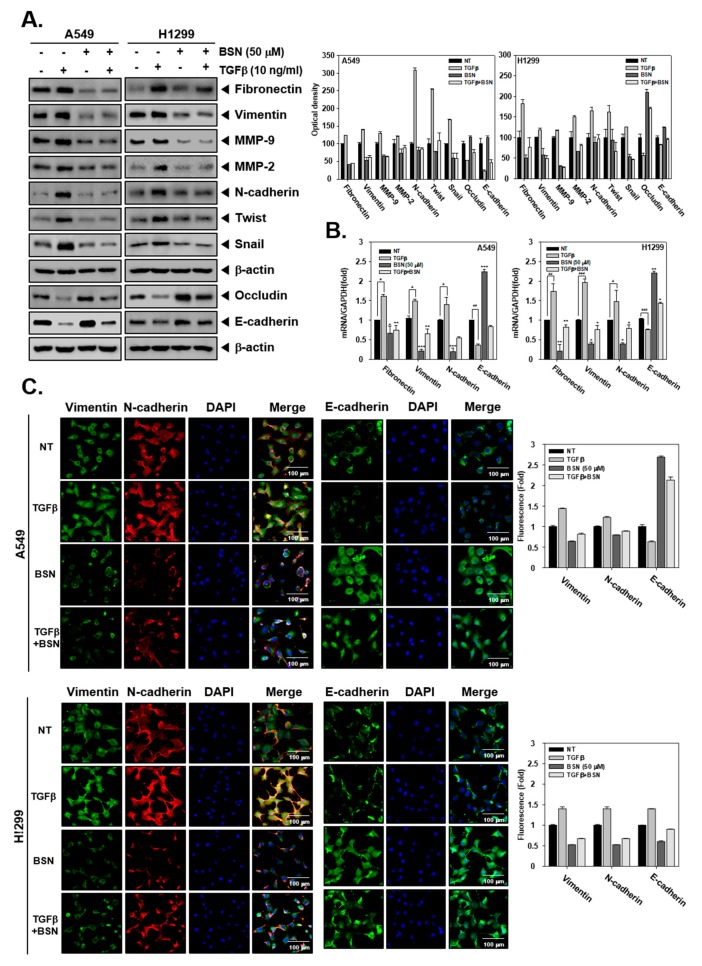
Effects of BSN on transforming growth factor beta (TGF-β) induced EMT process. (**A**) Cells were treated with TGF-β (10 ng/mL, −/+), BSN (50 μM, +/−), or the combination condition (+/+) for 24 h and western blotting was performed. The results shown are representative data of experiments. Graphs represent band intensities of indicated proteins. (**B**) Cells were treated as described above and real-time quantitative PCR was performed. Data represent means ± SD; ^###^ *p* < 0.001 vs. non-treated (NT) cells, ^##^ *p* < 0.01 vs. non-treated (NT) cells, ^#^ *p* < 0.05 vs. non-treated (NT) cells, *** *p* < 0.001 vs. TGF-β treated cells, ** *p* < 0.01 vs. TGF-β treated cells * *p* < 0.05 vs. TGF-β treated cells. (**C**) The cells were treated as described above and immunocytochemistry was carried out. Quantitative analysis of the fluorescence intensities was performed.

The levels of EMT markers were also examined in TGF-β-treated cells. As shown in Figure 2A, TGF-β exposure augmented expression of fibronectin, vimentin, MMP-9, MMP-2, N-cadherin, Twist, and Snail, while it downregulated occludin and N-cadherin compared with non-treated cells. BSN suppressed TGF-β-induced fibronectin, vimentin, MMP-9, MMP-2, N-cadherin, Twist, and Snail overexpression, whereas it substantially upregulated TGF-β-induced occludin and N-cadherin reduction. Also, Figure 2B revealed that identical changes in mRNA level were also observed as noted in EMT protein expression patterns. Moreover, immunocytochemistry data showed that vimentin and N-cadherin level increased and that of E-cadherin decreased in TGF-β-treated cells. BSN treatment also decreased TGF-β-stimulated vimentin and N-cadherin levels, whereas it increased the TGF-β-induced E-cadherin level (Figure 2C).

### 2.3. BSN Suppresses the Proliferation of Tumor Cells

**Figure 3:**
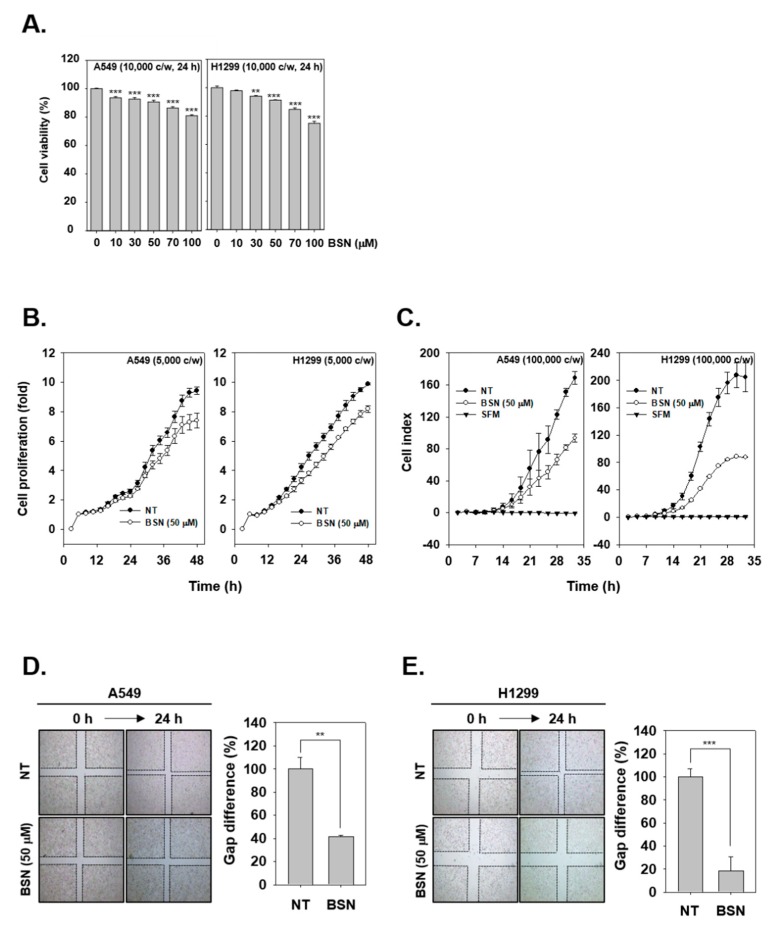
BSN represses various hallmarks of tumor cells. (**A**) A549 and H1299 cells (1 × 10^4^ cells/well) were treated with various doses of BSN and viability was examined. (**B**) BSN reduces the growth of tumor cells. (**C**) Invasive ability of cells was measured upon BSN treatment. (**D**,**E**) Anti-migratory potential was analyzed after exposure to BSN (50 μM) for 24 h. Data represent means ± SD; ** *p* < 0.01, *** *p* < 0.001.

To test the cytotoxic ability of BSN, we used MTT and the result showed that BSN exhibited less than 5% cytotoxicity in the cells up to 50 μM concentration (Figure 3A). Interestingly, BSN was also found to significantly attenuate cellular growth in the tested cell lines (Figure 3B).

### 2.4. BSN Inhibits Invasive and Migratory Capacity of Tumor Cells

To analyze the consequence of BSN treatment on the invasive ability of lung carcinoma cells, invasion assay was carried out. As shown in Figure 3C, BSN attenuated invasive activity in both A549 and H1299 cells. Next, we investigated the role of BSN in reducing the migratory ability of tumor cells, and as shown in Figure 3D,E, non-treated cells were noted to migrate significantly faster than BSN treated cells.

### 2.5. BSN Represses TGF-β-Induced Metastatic Effects

**Figure 4:**
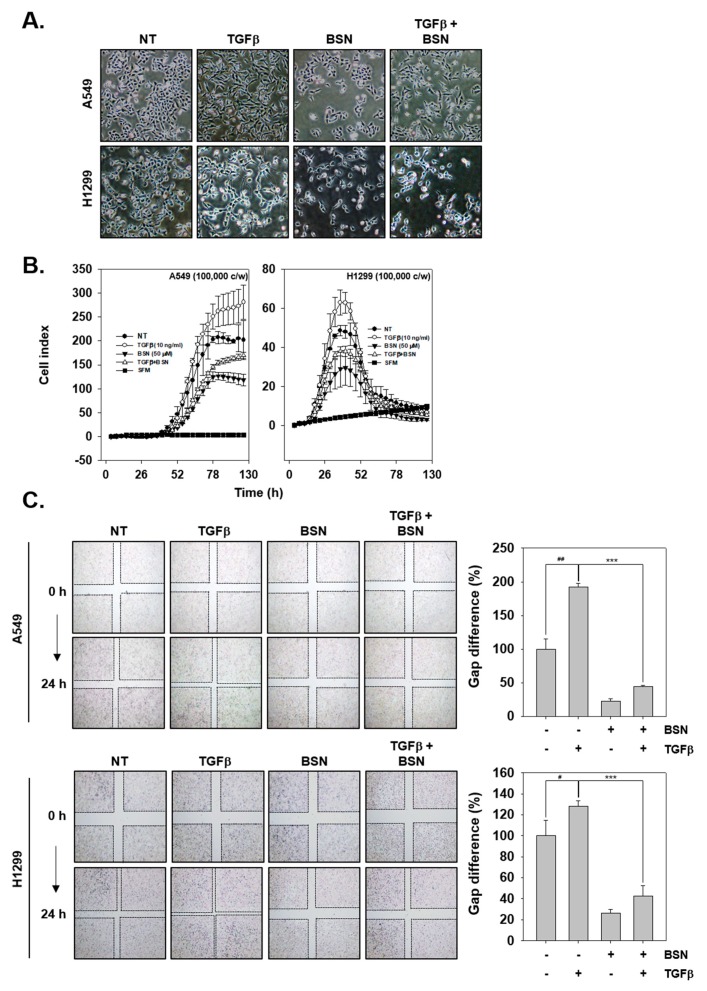
BSN treatment also modulates the oncogenic characteristics of TGF-β treated cells. (**A**) The cells were treated as described in Figure 2A and morphological changes were observed by Nikon ECLIPSE Ts2. (**B**) Invasion assay was performed as indicated in Figure 2. (**C**) Migration assay was carried out. Cells were treated with TGF-β (10 ng/mL, −/+), BSN (50 μM, +/−), or the combination condition (+/+) for 24 h. Data represent means ± SD; ^##^ *p* < 0.01 vs. non-treated (NT) cells, ^#^ *p* < 0.05 vs. non-treated (NT) cells, and *** *p* < 0.01 vs. TGF-β treated cells.

We also analyzed how BSN and TGF-β can affect EMT process. We took a picture of the possible morphological changes with TGFβ, BSN, or combination group of the two (Figure 4A). TGF-β-treated cells were found to be more elongated than non-treated cells and displayed a spindle like shape. However, BSN treatment prevented TGF-β-induced morphological changes, including spindle shaped morphology. In addition, we examined the role of BSN in reduction of TGF-β-activated cell invasion and migration. As shown in Figure 4B, TGFβ enhanced cell invasion, however, BSN was found to significantly down-regulate TGF-β-induced invasion. As shown in Figure 4C, wound healing assay was also investigated with TGF-β, BSN, or combination group. TGF-β-treated cells showed more wound closure and had less gap difference than non-treated cells. Moreover, BSN also inhibited TGF-β-induced migration in both lung tumor cells.

### 2.6. BSN Affects Activation of Multiple Oncogenic Molecules

**Figure 5:**
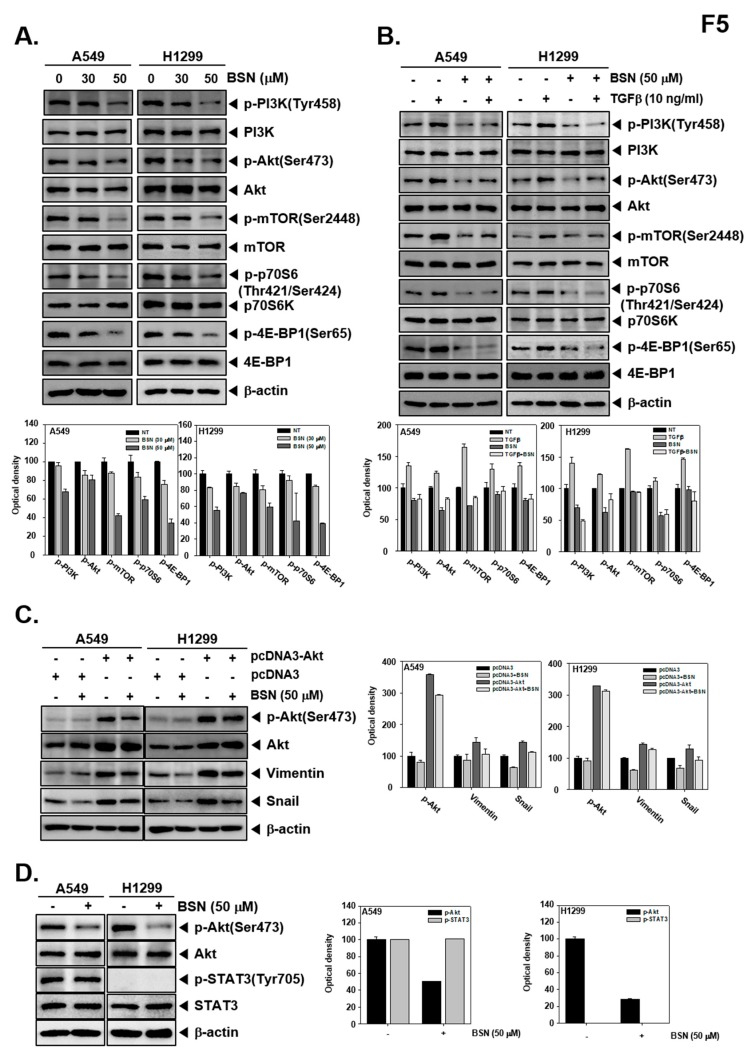
Effect of BSN on phosphoinositide-3-kinase (PI3K)/protein kinase B (Akt) in TGF-β-treated tumor cells. (**A**) Cells (5 × 10^5^ well) were exposed to BSN (30-50 μM) for 6 h and western blot was performed. (**B**) Three hours after exposure to BSN (50 μM, +/−), TGF-β (10 ng/mL, −/+), or the combination condition (+/+) was added for an additional three hours and western blotting was carried out. (**C**) A549 and H1299 cells were transfected with pcDNA3-Akt and pcDNA3 plasmids for 24 h. After that cells were treated with BSN 50 μM and Western blotting was performed. (**D**) Cells (5 × 10^5^ well) were treated with BSN (50 μM, +) for 6 h and Western blotting was performed. All the results shown are representative data of experiments. Graphs represent band intensities of indicated proteins.

We next investigated whether BSN modulated PI3K/Akt/mTOR/p70S6K/4E-BP1 phosphorylation in tumor cells. As shown in Figure 5A,B, BSN substantially downregulated both constitutive and TGF-β-induced Pl3K, Akt, mTOR, p70S6K, and 4E-BP1 activation. We examined whether overexpression of constitutive active Akt by pcDNA3-Akt plasmid can prevent the suppressive effect of BSN on EMT-related protein expression. First, A549 and H1299 cells were transfected with pcDNA3-Akt or pcDNA3 plasmid vectors. As shown in Figure 5C, BSN treatment in transfected cells slightly suppressed the phosphorylation of Akt, vimentin, and snail expression. Additionally, our previous study has demonstrated that treatment with BSN (200 μM) in A549 and H1299 cells suppressed the constitutive and inducible STAT3 signaling pathway [24]. Here, we found that treatment of BSN 50 μM inhibited the phosphorylation of Akt. However, BSN at 50 μM dose could not suppress both constitutive and inducible STAT3 activation (Figure 5D).

## 3. Discussion

This study deciphers the possible actions of BSN on the EMT-related cell signaling cascade. We found that BSN downregulated the levels of various mesenchymal markers and upregulated that of epithelial markers, and it can also substantially suppress TGF-β-induced fibronectin, vimentin, MMP-9, MMP-2, N-cadherin, Twist, and Snail expression, whereas it upregulated TGF-β-induced occludin and N-cadherin levels. Moreover, the suppression of EMT by BSN was found to cause an abrogation of proliferation and invasion. Interestingly, BSN inhibited not only constitutive but also inducible PI3K/Akt/mTOR/p70S6K/4E-BP1 phosphorylation in tumor cells.

EMT can cause loss of polarity in epithelial cells and downregulate the levels of epithelial markers, such as occluding, and then lead to the formation of mesenchymal cells via upregulation of the levels of markers, such as fibronectin, vimentin, etc. [27,28]. We observed for the first time that BSN downregulated fibronectin, vimentin, N-cadherin, Twist, and Snail levels at 50 μM concentration, while no toxic effect was detected at these concentrations. In addition, BSN substantially reduced the levels of the E-cadherin repressor proteins, Twist and Snail. Overall, findings suggest that the modulation of mesenchymal markers and epithelial markers by BSN can lead to blockage of EMT in lung carcinoma cells.

Matrix metalloproteinases (MMP)-9 and MMP-2 proteins can actively mediate the dissemination of tumor cells to distant sites [29,30]. Overexpression of MMP-9 and MMP-2 can also act as markers of metastasis and poor prognosis in patients [31,32]. We found that BSN substantially inhibited the MMP-9 and MMP-2 levels, thereby suggesting that the anti-metastatic effects displayed by BSN could be possibly mediated through the reduced activity of these two endopeptidases.

It was noted that this phytoalexin also suppressed the levels of upregulated mesenchymal markers and downregulated E-cadherin and occludin levels upon TGF-β treatment. We observed that BSN attenuated the conversion into a spindle-like morphology caused by TGF-β exposure Additionally, cellular invasion and migration promoted by TGF-β was attenuated by exposure to BSN in tumor cells.

**Figure 6:**
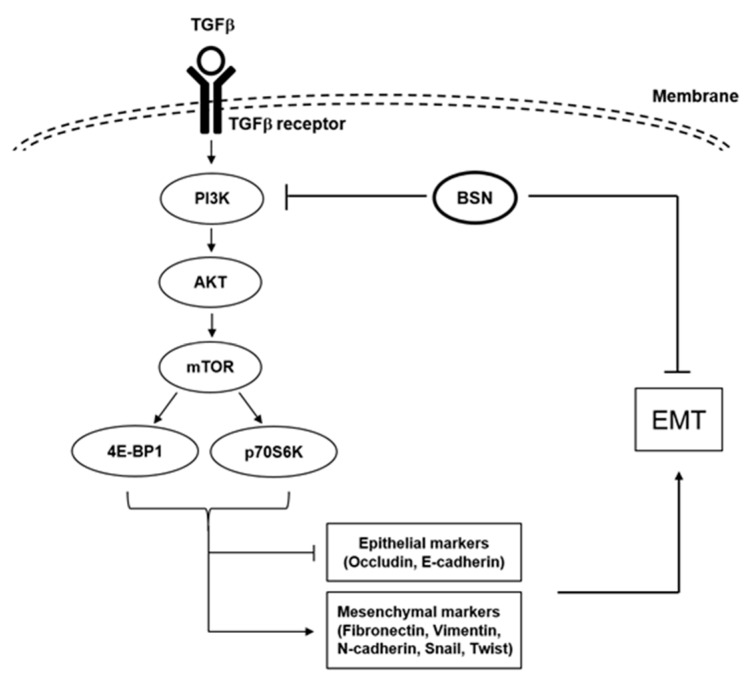
A diagrammatic representation of the possible actions of BSN on PI3K/Akt/mTOR cascade in tumor cells.

TGF-β has been found to cause induction of multiple oncogenic cascades, including PI3K, as well as Ras and Rho GTPases cascades [33], and thus drive the EMT process by controlling levels of diverse genes that can control metastasis. Our previous reports already suggest that PI3K/Akt/mTOR signaling cascade may have a crucial role in TGF-β-driven EMT phenomena [4,6]. In this study, we also found that BSN can substantially abrogate TGF-β-induced activation of these oncogenic kinases (Figure 6). However, additional experiments are required to decipher the different mechanisms underlying the beneficial actions of BSN against the metastatic spread of malignant cells.

## 4. Materials and Methods

### 4.1. Reagents and Cell Lines

Brassinin (BSN, Figure 1A) was purchased from LKT laboratories (Minneapolis, MN). Roswell Park Memorial Institute (RPMI) 1640, dulbecco’s modified eagle medium (DMEM) low glucose, fetal bovine serum (FBS), and penicillin-streptomycin mixture were purchased from Thermo Fisher Scientific Inc. (Waltham, MA, USA). The 3-(4,5-dimethylthiazol-2-yl)-2,5-diphenyltetrazolium bromide (MTT), Tris base, glycine, NaCl, sodium dodecylsulfate (SDS), and bovine serum albumin (BSA) were purchased from Sigma-Aldrich (St. Louis, MO, USA). Alexa Fluor^®^ 488 donkey anti-goat IgG (H+L) antibody and Alexa Fluor^®^ 594 donkey anti-rabbit IgG (H+L) antibody were obtained from Life Technologies (Grand Island, NY, USA). The iN-fect™ in vitro Transfection Reagent was obtained from iNtRON Biotechnology (Seongnam, Korea). Anti-Fibronectin, anti-Vimentin, anti-E-cadherin, anti-N-cadherin, anti-Occludin, anti-Twist, anti-MMP-2, anti-MMP-9, anti-Akt (all diluent, 1: 2000), and anti-β-actin antibodies (diluent, 1: 5000) were purchased from Santa Cruz Biotechnology (Santa Cruz, CA, USA). Anti-Snail, anti-p-PI3K(Tyr458), anti-PI3K, anti-p-Akt(Ser473), anti-p-mTOR(Ser2448), anti-mTOR, anti-p-p70S6K(Thr421/Ser424), anti-p70S6K, anti-p-4E-BP1(Ser65), and anti-4E-BP1 (all diluent, 1: 2000) antibodies were purchased from Cell Signaling Technology (Beverly, MA, USA). Human lung carcinoma cells (A549 and H1299 cells) were obtained from the American Type Culture Collection (Manassas, VA, USA). A549 cells were cultured in DMEM low glucose. H1299 cells were cultured in RPMI 1640 medium.

### 4.2. Western Blot Analysis

For detection of various antibodies, BSN-treated whole-cell extracts were lysed in a lysis buffer (20 mM Tris (pH 7.4), 250 mM NaCl, 2 mM EDTA (pH 8.0), 0.1% Triton X-100, 0.01mg/mL aprotinin, 0.005 mg/mL leupeptin, 0.4 mM phenyl methane sulfonyl fluoride (PMSF), and 4 mM NaVO4). Then, protein concentration in the whole cell lysates was measured by Bradford reagent (Bio-Rad, Hercules, CA, USA). An equal amount of lysates was resolved in an 8–15% SDS-polyacrylamide gel. After electrophoresis, the protein was transferred to the nitrocellulose membrane, blocked with 5% skim milk in 1× TBST (1× TBST with 0.1% Tween 20), and proved with specific primary antibodies: anti-Fibronectin, anti-Vimentin, anti-MMP-9, anti-MMP-2, anti-N-cadherin, anti-Twist, anti-Snail, anti-Occludin, anti-E-cadherin, anti-p-PI3K(Tyr458), anti-PI3K, anti-p-Akt(Ser473), anti-Akt, anti-p-mTOR(Ser2448), anti-mTOR, anti-p-p70S6K(Thr421/Ser424), anti-p70S6K, anti-p-4E-BP1(Ser65), and anti-4E-BP1. Antibodies were incubated at 4 °C overnight. Finally, membranes were washed with 1× tris buffered saline with tween 20 (TBST) and incubated with horseradish peroxidase (HRP) conjugated anti-rabbit IgG antibodies, anti-goat IgG antibodies, and anti-mouse IgG antibodies at room temperature for 2 h. The membranes were detected using chemiluminescence (ECL) (EZ-Western Lumi Femto, DOGEN) [4]. Densitometry values for Western blot experiments were estimated by Image J software (file version 1.4.3.67, National Institutes of Health, Bethesda, MD, USA).

### 4.3. Immunocytochemistry

A549 and H1299 cells were seeded in an 8-well glass chamber slide, and were treated with BSN for 24 h. Both cells were fixed with 4% paraformaldehyde (PFA) at room temperature for 20 min, washed three times with 1× phosphate-buffered saline (PBS), and permeabilized with 0.2% Triton-X-100. Then, they were blocked with 5% bovine serum albumin (BSA) in PBS for 1 h and incubated overnight at 4 °C with anti-Vimentin, anti-*N*-cadherin, and anti-E-cadherin (1:100; Santa Cruz, CA, USA). The next day, cells were washed three times by 1× PBS and incubated with Alexa Fluor^®^ 488 donkey anti-goat IgG (H+L) antibody and Alexa Fluor^®^ 594 donkey anti-rabbit IgG (H+L) antibody for 1 h at room temperature. The cells were stained with 4′,6-diamidino-2-phenylindole (DAPI) (1 μg/mL) for 3 min at room temperature and mounted on glass slides using Fluorescent Mounting Medium (Golden Bridge International Labs, Mukilteo, WA, USA). Finally, the fluorescence signal was detected by using an Olympus FluoView FV1000 confocal microscope (Tokyo, Japan) [4].

### 4.4. Real-Time Quantitative PCR

Total RNA was extracted with Trizol reagent and RNA was purified with chloroform and isopropanol. RNA was converted to cDNA and the expression levels of Fibronectin, Vimentin, N-cadherin, and E-cadherin were determined and compared using a StepOne real-time PCR instrument (Applied Biosystems, Foster City, CA, USA). Pairs of forward and reverse primer sets were used as follows: Fibronectin, 5′-ATGATGAGGTGCACGTGTGT-3′and 5′-CTCTGAATCATGGCATTGGT-3′. Vimentin, 5′-AGATGGCCCTTGACATTGAG-3′ and 5′-TGGAAGAGGCAGAGAAATCC-3′. N-cadherin, 5′-ATTGTGGGTGCGGGGCTTGG-3′ and 5′-GGGTGTGGGGCTGCAGATCG-3′. E-cadherin, 5′-TGCCCAGAAAATGAAAAAGG-3 and 5′-GTGTATGTGGCAATGCGTTC-3′.

### 4.5. 3-(4,5-Dimethylthiazol-2-yl)-2,5-Diphenyltetrazolium Bromide (MTT) Assay

Cell viability was measured using an MTT assay to detect nicotinamide adenine dinucleotide (NADH)-dehydrogenase activity. A549 and H1299 cells were treated with BSN (0, 10, 30, 50, 100 μM) for 24 h. After that, MTT solution (2 mg/mL) 30 μL was added into each well for 2 h and MTT lysis buffer 100 μL was added for overnight incubation. The absorbance was measured at 570 nm using an automated spectrophotometric plate reader. Cell viability was normalized as a relative percentage in comparison with non-treated controls [3].

### 4.6. Real-Time Cell Proliferation Analysis

Cell growth behavior was performed using the Roche xCELLigence Real-Time Cell Analyzer (RTCA) dual purpose (DP) instrument (Roche Diagnostics GmbH, Germany), as described previously. The cell growth pattern was determined as elaborated previously [4].

### 4.7. Invasion Assay

Instrument real-time monitoring of cellular invasion was performed using the Roche xCELLigence Real-Time Cell Analyzer (RTCA) DP instrument (Roche Diagnostics GmbH, Germany). The RTCA DP instrument used CIM (cellular invasion/migration)-plate 16 and measured electrical measurements. The upper chamber of the CIM-plates was coated with matrigel (BD Biosciences, Becton-Dickinson, Franklin Lakes, NJ, USA) and medium containing 10% FBS was placed in the lower chamber as a chemoattractant. Then, upper and lower chamber plates were assembled. Serum-free medium was placed in the upper chamber and incubated for 1 h at 37 °C and background impedance measurement was performed. After background measurement, A549 and H1299 cells (1 × 10^5^ cells/well) were seeded onto the upper chamber and the electrical impedance of the membrane was recorded every 15 min [4].

### 4.8. Wound Healing Assay

Wound healing assay was performed in the monolayers of cells. The cells (2 × 10^5^ cells/well) were plated in a 12-well plate and incubated until 80% confluence was reached. Then, the cell monolayer was scratched by a 200 μL micropipette tip and washed with serum-free medium. Next, cells were treated with BSN and incubated with serum-free medium for 24 h. The width of the wound was observed by using a microscope (Nikon ECLIPSE Ts2., Tokyo, Japan) at time 0 and 24 h. Gap distance of the wound was measured at four different sites and non-treated samples were used as controls [4].

### 4.9. Transfection with pcDNA3-myr-Ha-Akt1 Plasmid.

The iN-fect™ in vitro Transfection Reagent (iNtRON Biotechnology, Seongnam, Korea) was used for transfection with Akt expression vectors (Adgene plasmid 9008; pcDNA3-myr-Ha-Akt1). A549 and H1299 cells were transfected with pcDNA3-Akt (300 ng) or pcDNA3 (300 ng) for 24 h in serum-free media. After transfection, cells were treated with BSN for 6 h. The cell lysates were prepared for Western blot analysis.

### 4.10. Statistical Analysis

All experiments are presented as the mean ± standard deviation (SD). Statistical significance was analyzed by Mann-Whitney U test.
